# Indirect Routes to Aminoacyl-tRNA: The Diversity of Prokaryotic Cysteine Encoding Systems

**DOI:** 10.3389/fgene.2021.794509

**Published:** 2022-01-03

**Authors:** Takahito Mukai, Kazuaki Amikura, Xian Fu, Dieter Söll, Ana Crnković

**Affiliations:** ^1^ Department of Molecular Biophysics and Biochemistry, Yale University, New Haven, CT, United States; ^2^ Department of Life Science, College of Science, Rikkyo University, Tokyo, Japan; ^3^ Department of Chemistry, Yale University, New Haven, CT, United States; ^4^ Department of Molecular Biology and Nanobiotechnology, National Institute of Chemistry, Ljubljana, Slovenia

**Keywords:** aminoacyl-tRNA synthetases, *O*-phosphoseryl-tRNA synthetase, genetic code, tRNA, cysteine, selenocysteine, bioinformatics, metagenome

## Abstract

Universally present aminoacyl-tRNA synthetases (aaRSs) stringently recognize their cognate tRNAs and acylate them with one of the proteinogenic amino acids. However, some organisms possess aaRSs that deviate from the accurate translation of the genetic code and exhibit relaxed specificity toward their tRNA and/or amino acid substrates. Typically, these aaRSs are part of an indirect pathway in which multiple enzymes participate in the formation of the correct aminoacyl-tRNA product. The indirect cysteine (Cys)-tRNA pathway, originally thought to be restricted to methanogenic archaea, uses the unique *O*-phosphoseryl-tRNA synthetase (SepRS), which acylates the non-proteinogenic amino acid *O*-phosphoserine (Sep) onto tRNA^Cys^. Together with Sep-tRNA:Cys-tRNA synthase (SepCysS) and the adapter protein SepCysE, SepRS forms a transsulfursome complex responsible for shuttling Sep-tRNA^Cys^ to SepCysS for conversion of the tRNA-bound Sep to Cys. Here, we report a comprehensive bioinformatic analysis of the diversity of indirect Cys encoding systems. These systems are present in more diverse groups of bacteria and archaea than previously known. Given the occurrence and distribution of some genes consistently flanking SepRS, it is likely that this gene was part of an ancient operon that suffered a gradual loss of its original components. Newly identified bacterial SepRS sequences strengthen the suggestion that this lineage of enzymes may not rely on the m^1^G37 identity determinant in tRNA. Some bacterial SepRSs possess an N-terminal fusion resembling a threonyl-tRNA synthetase editing domain, which interestingly is frequently observed in the vicinity of archaeal SepCysS genes. We also found several highly degenerate SepRS genes that likely have altered amino acid specificity. Cross-analysis of selenocysteine (Sec)-utilizing traits confirmed the co-occurrence of SepCysE and the Sec-utilizing machinery in archaea, but also identified an unusual *O*-phosphoseryl-tRNA^Sec^ kinase fusion with an archaeal Sec elongation factor in some lineages, where it may serve in place of SepCysE to prevent crosstalk between the two minor aminoacylation systems. These results shed new light on the variations in SepRS and SepCysS enzymes that may reflect adaptation to lifestyle and habitat, and provide new information on the evolution of the genetic code.

## 1 Introduction

**FIGURE 1:**
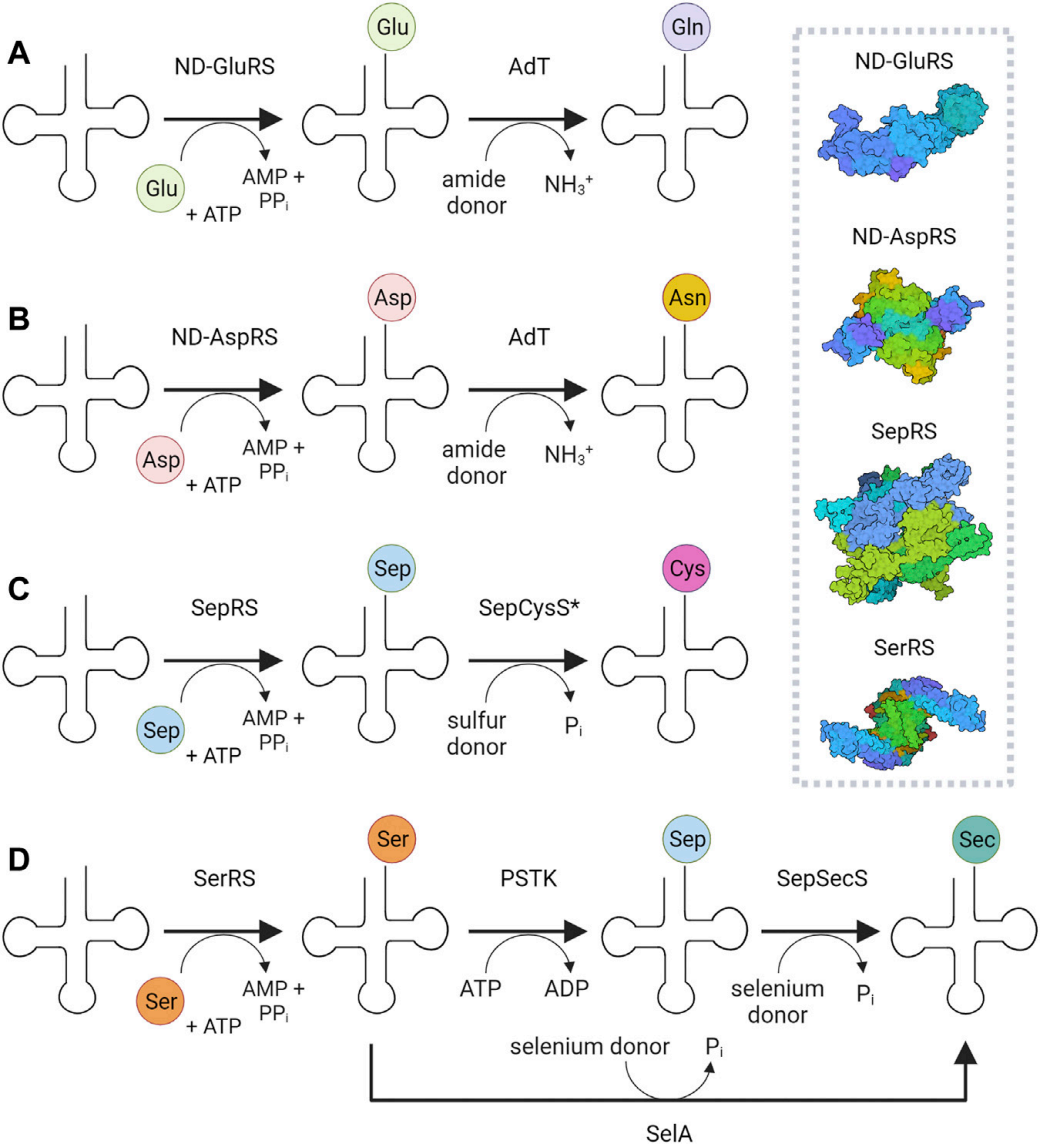
Indirect pathways to glutaminyl-**(A)**, asparaginyl-**(B)**, and cysteinyl-tRNAs **(C)**, and selenocysteinyl-tRNA **(D)**. The surface representation of the corresponding aaRSs is shown: ND-GluRS—non-discriminating glutaminyl-tRNA synthetase (PDB id: 2CFO) (Schulze et al., 2006); ND-AspRS—non-discriminating aspartyl-tRNA synthetase (PDB id: 1N9W) (Charron et al., 2003); SerRS—seryl-tRNA synthetase (PDB id: 4L87) (Xu et al., 2013); SepRS—*O*-phosphoseryl-tRNA synthetase (PDB id: 2ODR) (Kamtekar et al., 2007). AdT—amidotransferase, SepCysS—Sep-tRNA:Cys-tRNA synthase, PSTK—*O*-phosphoseryl-tRNA^Sec^ kinase, SepSecS—Sep-tRNA:Sec-tRNA synthase, SelA—selenocysteine synthase A. The asterisk "*" indicates that SepCysE may be present. In 1D, the eukaryotic and archaeal pathway for Sec-tRNA biosynthesis is shown; in bacteria, a single enzyme, SelA, carries out the conversion of Ser to Sec, as indicated by the arrow below.

The essentially universal presence of twenty proteinogenic canonical amino acids in organismal proteomes is manifested by the existence of one or more aminoacyl-tRNA synthetase (aaRS) genes for each amino acid. These enzymes embody the major aminoacylation systems that generate by direct acylation the correctly charged (cognate) aminoacyl-tRNA (aa-tRNA) species for ribosomal protein synthesis (Yuan et al., 2008; Rubio Gomez and Ibba, 2020). Yet additional routes to aa-tRNA formation exist (Sheppard et al., 2008) (Figure 1). These indirect pathways rely on non-discriminating aaRSs that form a misacylated tRNA intermediate, whose tRNA-bound non-cognate amino acid is subsequently converted by a different enzyme to the desired cognate aa-tRNA (Figure 1).There is thus a fundamental difference between aaRSs of the direct and indirect pathways to aa-tRNA. The major aminoacylation systems rely on *discriminating* aaRSs that rigorously match the amino acid to its cognate tRNA, and thus ensure faithful translation of the genetic code. In contrast, the indirect translation systems depend on *non-discriminating* aaRSs that form non-cognate aa-tRNAs, whose misacylated amino acids are converted to the correct amino acid substrate by a set of different enzymes. These minor aminoacylation systems are the sole route to asparaginyl (Asn)-, glutaminyl (Gln)-, cysteinyl (Cys)-tRNA in many prokaryotic organisms or organelles; the RNA-dependent selenocysteine (Sec) biosynthesis produces Sec-tRNA in all three domains of life. With the exception of Cys-tRNA synthesis, each of these indirect pathways starts with an aaRS of relaxed specificity: non-discriminating aspartyl- (ND-AspRS) and glutamyl-tRNA synthetases (ND-GluRS) attach aspartate (Asp) and glutamate (Glu) to tRNA^Asn^ and tRNA^Gln^, respectively, while seryl-tRNA synthetase (SerRS) ligates serine (Ser) to tRNA^Sec^ (Figure 1). Thus, the three aaRSs belonging to these minor acylation systems show relaxed specificity towards tRNA. The aaRS devoted to indirect Cys-tRNA synthesis, *O*-phosphoseryl-tRNA synthetase (SepRS), is unique in a sense that it does not ligate a proteinogenic amino acid, but the serine biosynthetic pathway intermediate (Helgadóttir et al., 2007) *O*-phosphoserine (Sep) to tRNA^Cys^.

**FIGURE 2:**
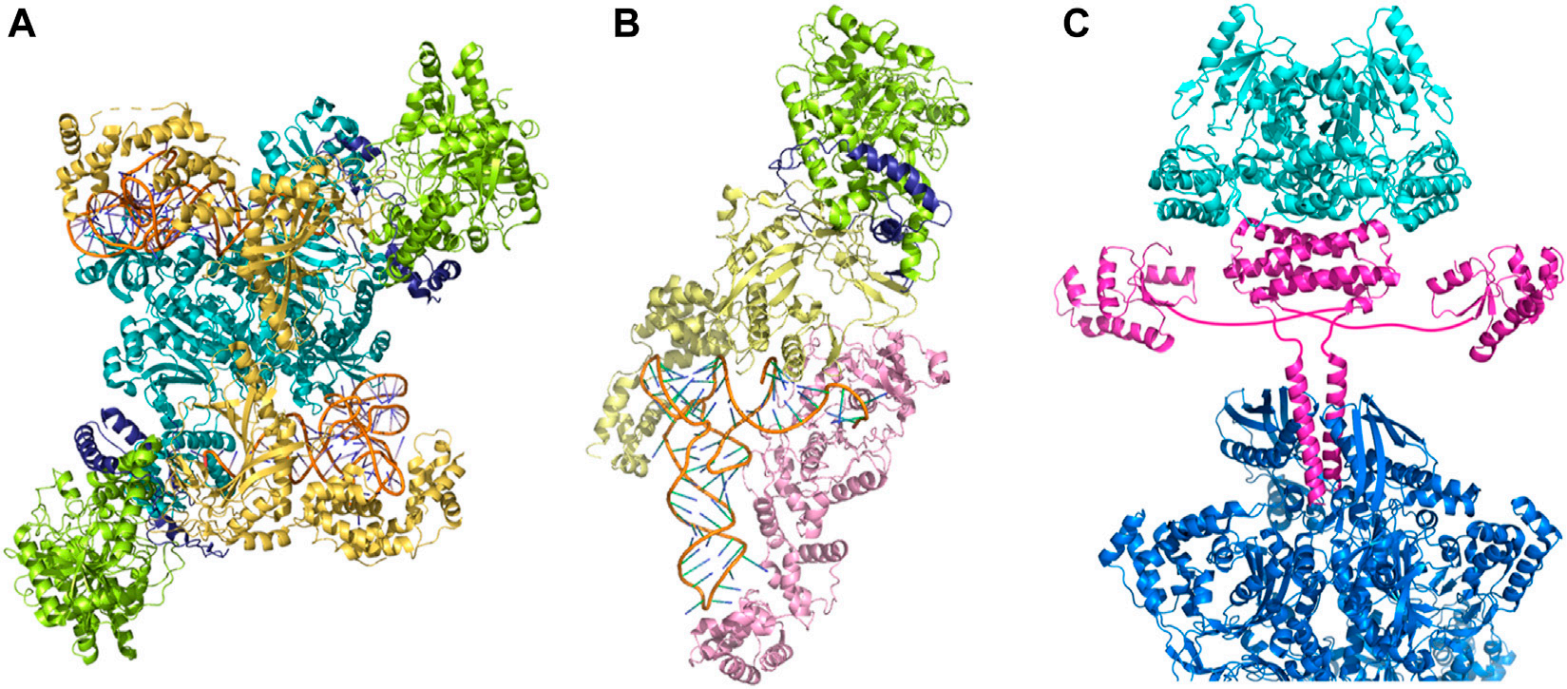
Macromolecular complexes embody the indirect pathway to aminoacyl-tRNAs. **(A)** Asparaginyl-transamidosome from *Pseudomonas aeruginosa* (Suzuki et al., 2015) (PDB id: 4WJ3), **(B)** glutaminyl-transamidosome from *Thermotoga maritima* (Ito and Yokoyama, 2010) (PDB id: 3AL0), **(C)** a model of the transsulfursome from *Methanocaldococcus jannaschii* (Chen et al., 2017b) (PDB ids: 5X6B, 5X6C). Amidotransferase subunits are shown in light green (GatA), yellow (GatB), and dark blue (GatC); ND-AspRS is shown in teal, and ND-GluRS in light pink. One half of tetrameric SepRS is visible and shown in blue; SepCysS and SepCysE are shown in cyan and magenta, respectively.

Only a handful of prokaryotic organisms encodes a full complement of aaRSs (Sheppard et al., 2008; Chaliotis et al., 2017). GlnRS is absent from most bacterial and archaeal genomes (Chaliotis et al., 2017; Di Giulio, 2020), whereas AsnRS is mainly absent in archaea and to a lesser extent in bacteria (Di Giulio, 2020). The relaxed specificity of ND-AspRS and ND-GluRS allows these synthetases to acylate tRNA^Asn^, and tRNA^Gln^, respectively. To maintain translational accuracy, the misacylated tRNAs are shuttled directly to the Asp/Glu-amidotransferase (AdT) via the formation of the transamidosome complex (Figures 2A,B); the amidotransferase then converts the tRNA-bound Asp/Gln to Asn/Gln, resulting in correctly acylated tRNA. The heterotrimeric amidotransferase GatCAB is found in bacteria and archaea, while the dimeric amidotransferase GatDE exists exclusively in archaea, and is considered an archaeal signature protein (Sheppard and Söll, 2008). In both instances, the reaction proceeds in the same manner: the side-chain carboxyl group of Glu or Asp is first activated by phosphorylation and then amidated using the ammonia released from an amide donor (Figure 1).

**FIGURE 3:**
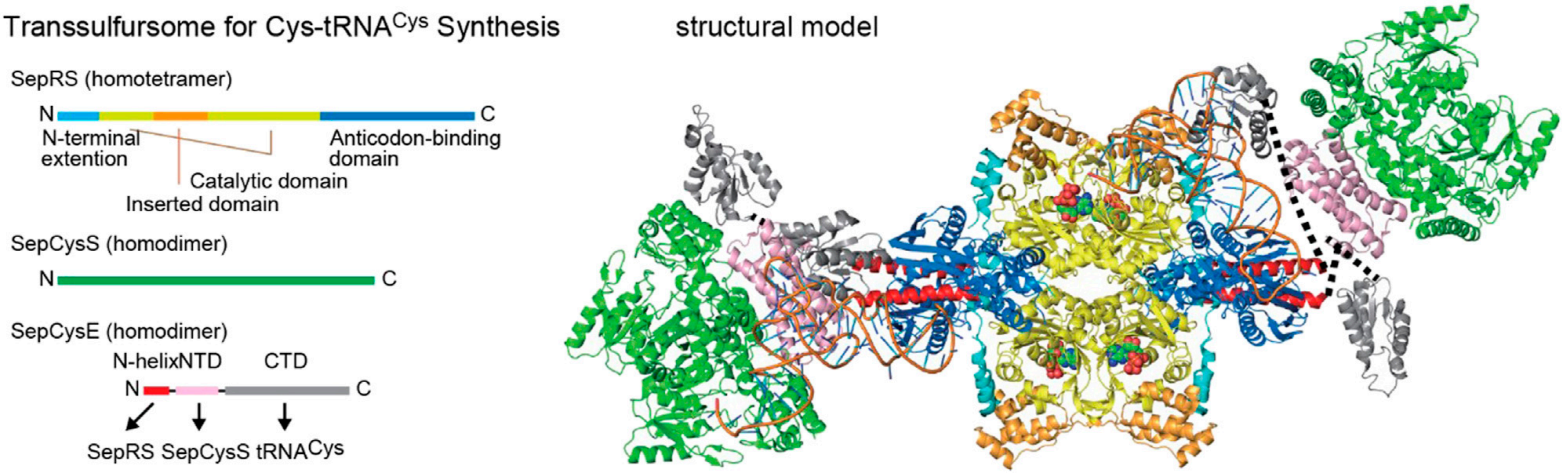
Components and structural model of a transsulfursome [adapted from references (Chen. et al., 2017b) and (Fukunaga and Yokoyama, 2007)] with a color scheme depicting the domain structures. Dotted lines represent flexible linkers between the two SepCysE domains.

Many methanogens lack cysteinyl-tRNA synthetase (CysRS) and use the indirect pathway for Cys-tRNA synthesis, a route used for both Cys biosynthesis and encoding (Sauerwald et al., 2005). This two-step process begins with the acylation of tRNA^Cys^ with Sep, catalyzed by SepRS. Subsequently, Sep-tRNA^Cys^ is converted to Cys-tRNA^Cys^ by Sep-tRNA:Cys-tRNA synthase (SepCysS) (Figure 1). The transsulfursome complex includes both enzymes plus tRNA^Cys^ and, in the case of class I methanogens and some other archaea, a SepCysE adapter protein (Liu et al., 2014; Mukai et al., 2017a). SepCysE has a high affinity for both SepRS and SepCysS and enables the formation of a stable SepRS•SepCysS•SepCysE•tRNA^Cys^ complex, named the transsulfursome (Liu et al., 2014; Chen et al., 2017b) (Figures 2, 3). Importantly, some SepRS-containing archaea lack one or all enzymes of the general Cys biosynthetic pathway and rely on SepRS-mediated Cys-tRNA formation; free Cys is then generated by Cys-tRNA deacylation or protein turnover (Sauerwald et al., 2005).

In all organisms, the pathway to Sec-tRNA^Sec^ begins with SerRS, but the conversion of the Ser moiety to Sec is catalyzed by different enzymes. In bacteria, selenocysteine synthase SelA catalyzes the conversion of Ser to Sec (Leinfelder et al., 1988) (Figure 1). However, a two-step process is found in Sec-utilizing archaea and eukaryotes, where the tRNA-bound serine is first phosphorylated by *O*-phosphoseryl-tRNA^Sec^ kinase (PSTK) to Sep-tRNA^Sec^. The misacylated tRNA is subsequently converted to Sec-tRNA^Sec^ by Sep-tRNA:Sec-tRNA synthase (SepSecS) (Yuan et al., 2006; Xu et al., 2007) (Figure 1).

As illustrated by the transamidosome and transsulfursome structures, the macromolecular complexes comprising multiple components of the pathway embody the concept of minor acylation systems (Figure 2). Complex formation allows substrate tunneling that 1) prevents release of misacylated tRNA to the protein synthesizing machinery, thus ensuring the fidelity of genetic code translation; and 2) enables efficient shuttling of intermediates between different enzymes of the pathway. Analogous to indirect Asn-, Gln- and Cys-tRNA synthesis, Sec-tRNA^Sec^ biosynthesis may also be facilitated by a PSTK•SepSecS•tRNA^Sec^ complex. This highlights another unusual feature of these four encoding pathways: the propensity to function as biosynthetic pathways for their corresponding amino acids.

We have previously shown that the two-step Cys biosynthesis and encoding pathway is not restricted to methanogenic archaea, but occurs in various archaeal as well as some bacterial clades (Mukai et al., 2017a). Cross-analysis with Sec-utilizing traits in these organisms revealed that the adapter protein SepCysE is present only in Sec-utilizing archaea, suggesting coevolution of the two minor acylation systems. It is important to note that Sec-tRNA^Sec^ is also synthesized via a Sep-tRNA intermediate in archaea and eukaryotes (Yuan et al., 2006; Xu et al., 2007; Rother and Quitzke, 2018; Mariotti et al., 2019) (Figure 1). In archaea that do not use Sec, and therefore lack SepCysE, SepCysS is often accompanied by or fused to a small peptide homologous to the SepCysS-binding domain of SepCysE (‘SepCysSn’ as a standalone peptide, or ‘SepCysSN’ when fused to SepCysS) (Mukai et al., 2017a). The present study arose from our desire to perform a thorough analysis of two minor aminoacylation systems by searching all sequence data available in the public databases as a follow-up to our earlier work (Mukai et al., 2017a). Here, we confirm that host groups/species were successfully annotated in almost all cases. Furthermore, multiple examples were identified for each minor group of bacteria and archaea and several attractive groups of archaea, such as new groups of methanogens, DPANN groups, and Asgard groups, were analyzed. By analyzing the distribution and divergence of minor Cys- and Sec-encoding systems, we aim to lay the foundation for the discovery of new metabolic pathways and conditions that determine the broader range of activities characteristic of aaRSs belonging to the minor acylation systems.

## 2 Materials and Methods

### 2.1 Bioinformatics

The public webserver-based BLASTp, BLASTn, tBLASTn, and SRA BLAST tools of the Integrated Microbial Genomes & Microbiome (IMG/M) system (Chen I.-M. A. et al., 2017) and of NCBI were used for bioinformatic analyses, as described previously (Mukai et al., 2017a). The NCBI SRA tool kit ver. 2.9.6-1-win64 was used for the manual assembly and curation of SepRS-like sequences from SRA data. Multiple alignment analyses were performed by using Clustal X 2.1 (Larkin et al., 2007) and Seaview 4.5.4 (Gouy et al., 2010) followed by manual curation based on the crystal structural information. Phylogenetic unrooted trees were developed by maximum likelihood estimation with 100 replicates using MEGA-X (Kumar et al., 2018) by using the Maximum Likelihood method based on the JTT matrix-based model (bootstrap method, uniform rates, use all sites). Tree manipulations were done with FigTree v1.4.3.3D modeling and rendering of proteins and tRNAs was performed with PyMol 1.7.6.0 (Schrödinger, LLC). Figures were created using Adobe Illustrator and Biorender.com. This update of our previous annotations (Mukai et al., 2017a) was done on October 5, 2021.

## 3 Results

### 3.1 Refining the Phylogenetic Trees of SepRS, SepCysS, and SepCysE Homologs

Our previous analysis has shown that the components of the indirect Cys encoding pathway are present in organisms beyond euryarchaeal methanogens, including TACK, Asgard and DPANN archaeal superphyla, as well as some bacteria (Mukai et al., 2017a). A putative ancestral operon was also discovered in which the SepRS gene is accompanied by translin-associated protein X (TRAX). This current analysis, performed using newly deposited genome and metagenome datasets in the NCBI and JGI IMG/M (Supplementary Table S1) in combination with the protein sequences used for our previous phylogenetic analysis, confirms our earlier results and shows that the two-step Cys encoding pathway is even more widespread than previously thought (Mukai et al., 2017a).

#### 3.1.1 Features of Newly Identified Bacterial and Archaeal Two-step Cys-Encoding Systems

**FIGURE 4:**
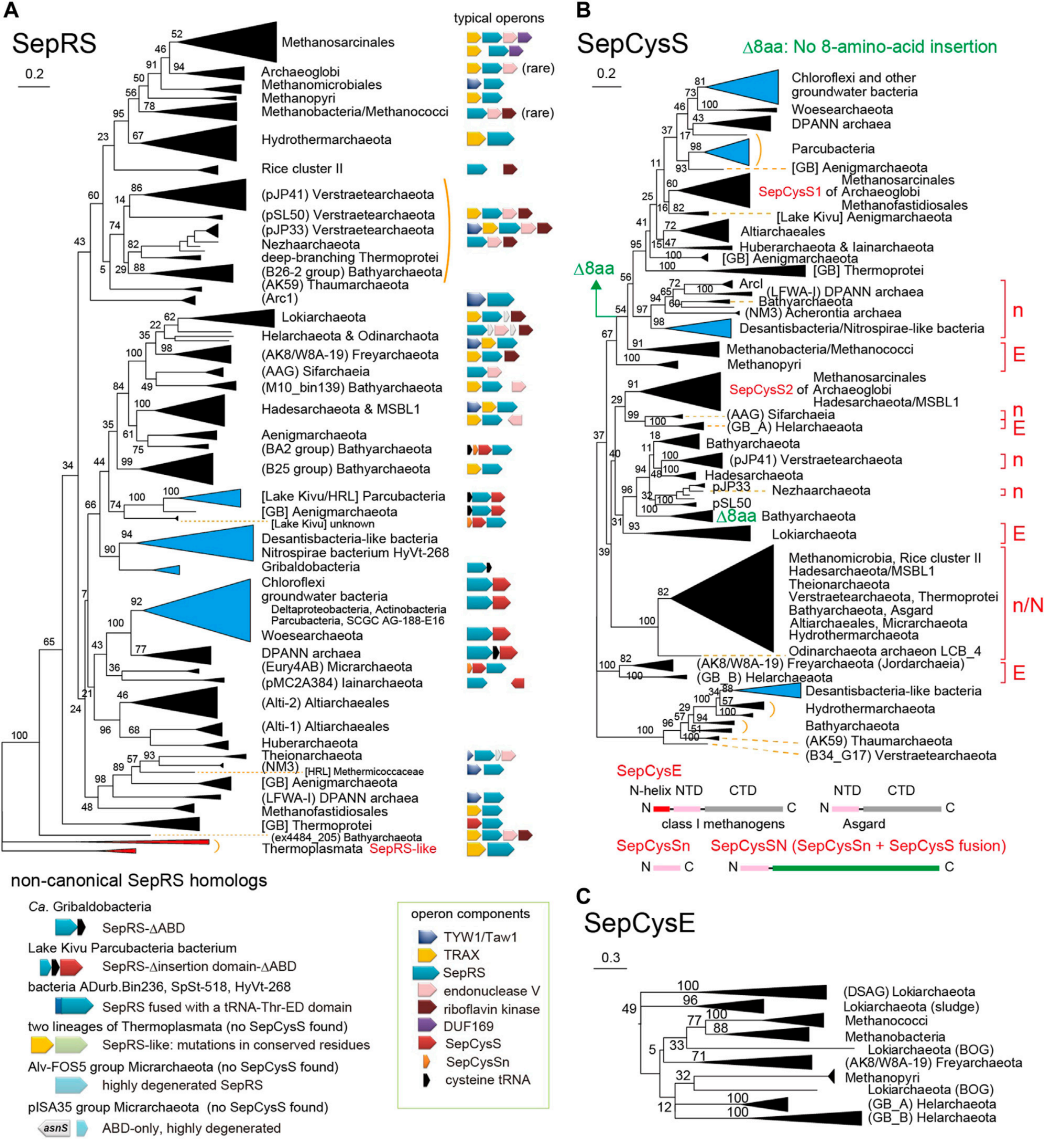
Phylogenetic trees of SepRS **(A)**, SepCysS **(B)**, and SepCysE **(C)** (updated from Mukai et al., 2017a). These unrooted trees were made by maximum likelihood estimation with 100 replicates. The bootstrap values (percentages) are shown. The SepRS and SepCysS clades consisting of or containing bacterial homologs are presented with blue triangles. In the SepCysS tree, genetic association of SepCysS with SepCysE (E) or SepCysSn (n) or fusion with SepCysSn (N) are indicated. The lack of the eight-amino-acid insertion near the catalytic site of SepCysS species (Mukai et al., 2017a) is specified by green arrows. For simplicity, the interim taxonomic status for uncultured prokaryotes is not denoted with “*Candidatus*” and “*Ca.*“. In some cases, metagenomic origins (Lake Kivu, HRL, activated sludge, BOG ECP12_OM1, and Guaymas Basin) are given.

The updated phylogenetic trees of SepRS, SepCysS, and SepCysE reveal several new features (Figure 4). 1) SepRS and SepCysS exist in a few bacterial and some obscure archaeal lineages (Figures 4A,B). 2) Although SepCysE is not present in bacterial lineages, the peptide homolog of its N-terminal domain, SepCysSn, has been found in a few bacteria. 3) SepCysE is present in several lineages of Asgard archaea (Bulzu et al., 2019; Seitz et al., 2019; Imachi et al., 2020) (Figure 4C, Supplementary Figure S1); however, the N-terminal helix of SepCysE homologs responsible for SepRS binding in class I methanogens (Chen M. et al., 2017) (Figure 3 and Figure 4B) was found to be absent in the N-terminal domain of SepCysE in Asgard archaea (Figure 4B). This could indicate the absence of the transsulfursome complex (Figure 3) (Chen M. et al., 2017) in Asgard archaea, where SepRS might be detached from the SepCysE-SepCysS complex.

#### 3.1.2 SepRS Is Part of Two Distinct Gene Clusters in Different Lineages

Some clear trends can be observed in the updated phylogenetic trees. In the Euryarchaeota and the superphyla TACK and Asgard, the SepRS gene is frequently flanked by particular genes in the same operon or gene cluster (Figure 4A). Most commonly, SepRS sequences are accompanied by genes encoding TRAX and endonuclease V. Occasionally, TYW1/Taw1, riboflavin kinase, or DUF169 are also present. Some hydrothermal vent lineages of *Ca.* Bathyarchaeota, deeply branching Thermoprotei, and *Ca.* Verstraetearchaeota archaea (Dombrowski et al., 2018) have the ‘most complete’ operon. Therefore, it is tempting to assume a gradual loss of operon components in the other lineages. In contrast, the SepRS sequences of DPANN archaea, early branching Euryarchaeota, and bacteria often form a single operon with genes encoding SepCysS and sometimes tRNA^Cys^ (Figure 4A).

### 3.2 Diversity of Bacterial SepRS Species

#### 3.2.1 Divergent Composition of Anticodon Binding Domains of Bacterial SepRSs

**FIGURE 5:**
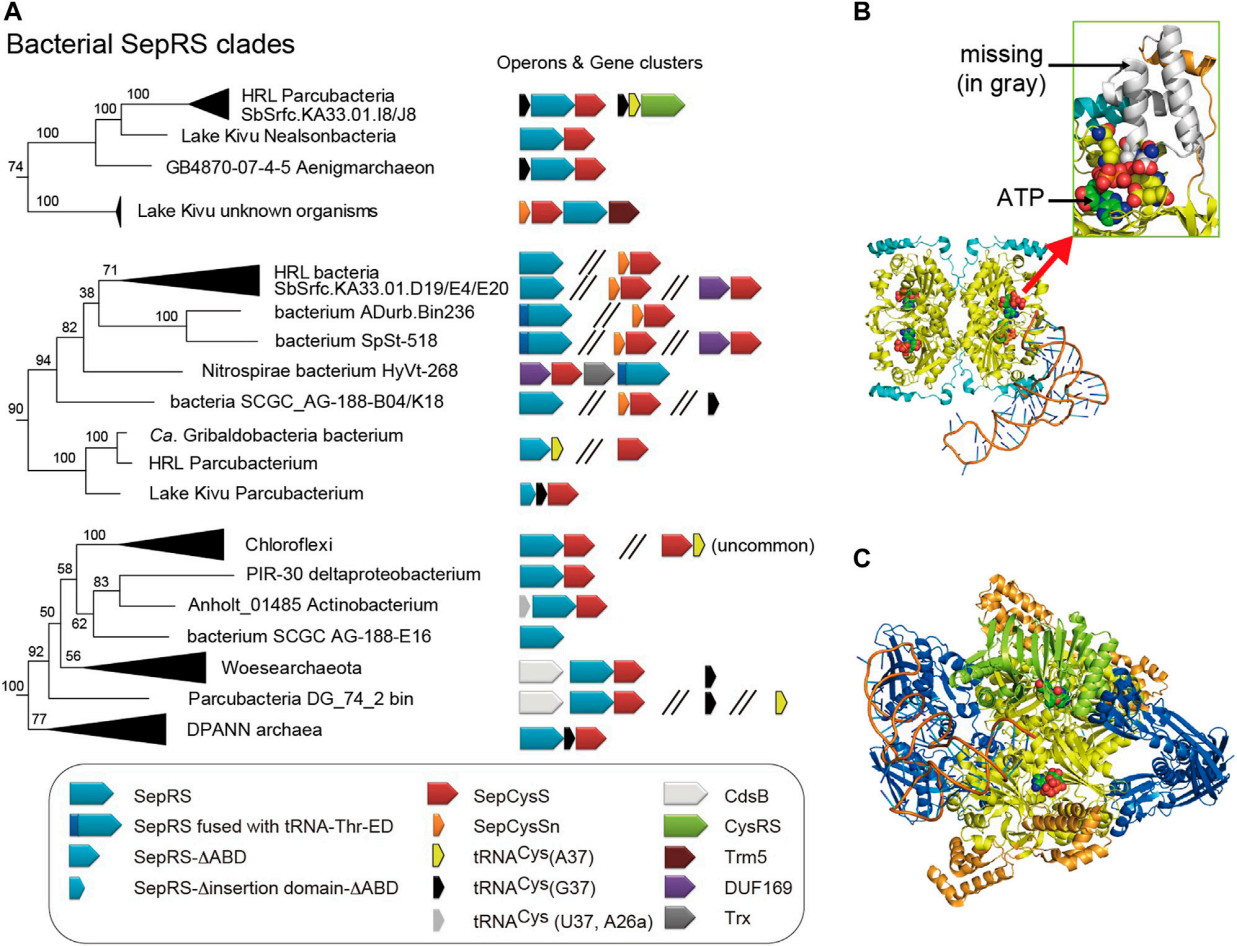
Diversity of bacterial SepRS species. **(A)** The terminal branches of the three bacterial SepRS clades from the phylogenetic tree of Figure 4A are shown, together with the operon structures of their SepRS and SepCysS genes. Some of these bacteria may possess tRNA^Cys^ with G37 which is uncommon in bacteria but is recognized by archaeal SepRS via the m^1^G37-recognizing motif. A few Parcubacteria strains may possess a SepRS lacking the anticodon binding domain (ABD). One SepRS variant further lacks most part of the insertion domain. Three SepRS variants were N-terminally fused via a 20-amino-acid linker with a “tRNA-Thr-ED” homolog which corresponds to the serine editing domain of archaeal ThrRS. Hypothetical models of these exceptional SepRS **(B,C)** follow the same color scheme as in Figure 3B. A model of bacterial SepRS lacking the insertion and anticodon binding domains in complex with cognate tRNA. The model shows that both tRNA (anticodon, acceptor stem) and ATP recognition (magnified in the green rectangle) may be impaired **(C)** A model of SepRS fused to the editing domain of ThrRS. The acceptor stem of tRNA can move between the synthetic active site of SepRS and the hydrolytic (editing) site of the editing domain. The tRNA-Thr-ED model (green) is from the crystal structure of the editing domain of *M. jannaschii* ThrRS with the substrate analog l-Ser3AA (PDB id: 4RRF).

The updated phylogeny reveals four clades of SepRS homologs in the bacterial domain (Figure 4A, 5A). It is important to note that SepRS, whose closest evolutionary relative is phenylalanyl-tRNA synthetase (PheRS) rather than CysRS (O'Donoghue et al., 2005), uses the same, specific set of identity elements as CysRS, including the GCA anticodon, the U73 discriminator base, and the methylated G37 (m^1^G37) base (Zhang et al., 2008). In most archaea and eukaryotes, the m^1^G37 base in tRNA^Cys^ serves to increase the efficiency of aminoacylation for both SepRS and CysRS (Zhang et al., 2008; Goto-Ito et al., 2017). However, bacterial SepRS lacking the m^1^G37-recognition motif can acylate tRNA^Cys^ with A37 or unmodified G37 *in vitro* (Mukai et al., 2017a), consistent with the fact that most bacteria have tRNA^Cys^ with A37. Newly identified, even larger truncations of the anticodon binding domain (ABD) containing the m^1^G37-recognition motif are present in some SepRSs from Parcubacteria (Figures 5A,B); this may suggest that base 37 is irrelevant for bacterial SepRS enzymes. In contrast, some of the SepRS species in the recently identified bacterial SepRS clades retain the m^1^G37 recognition motif and are coupled to tRNA^Cys^ (G37) (Figure 5A).

#### 3.2.2 Bacterial SepRS Enzymes Fused to an N-Terminal Editing Domain

The SepRSs of some Desantisbacteria-like bacteria and Nitrospirae bacterium HyVt-268 lack the canonical N-terminal extension and are instead equipped with a domain resembling an archaeal editing domain on their N-termini. This domain has all hallmarks of a serine editing domain of archaeal threonyl-tRNA synthetase (ThrRS) (Korenčić et al., 2004; Ahmad et al., 2015) (Figure 5C). Although the ThrRS editing domain (tRNA-Thr-ED) is usually fused to the ABD in ThrRS-ED or to both the ABD and aminoacylation domains in archaeal full-length ThrRS (Korenčić et al., 2004; Ahmad et al., 2015), the tRNA-Thr-ED domain alone has the ability to discriminate tRNA species (Novoa et al., 2015), and relies on the U73 discriminator base of archaeal tRNA^Thr^. Thus, although the ThrRS editing domain recognizes misacylated tRNA^Thr^, it could also hydrolyze misacylated tRNA^Cys^, since this tRNA also contains the U73 determinant. Structural modeling of a SepRS-tRNA-Thr-ED fusion protein shows that the fused editing domain forms a homodimer near the SepRS aminoacylation sites (Figure 5C). This type of configuration would allow the flexible CCA end of tRNA^Cys^ to move between the synthetic and editing sites of the fusion enzyme. Interestingly, the serine recognition motif of tRNA-Thr-ED is completely conserved, raising the question of the identity of amino acid that is inaccurately ligated to bacterial tRNA^Cys^ in the first place.

As demonstrated previously, a standalone tRNA-Thr-ED homolog is often associated with a SepCysS gene belonging to some euryarchaeal and DPANN species (SepCysS clade VII (Mukai et al., 2017a),). It is also present in the crenarchaeon *Ignicoccus hospitalis* KIN4/I (and some other uncultured Crenarchaea species) and is annotated as Ser-tRNA^Thr^ hydrolase (WP_011,998,431) (Podar et al., 2008). The reason why in bacteria this tRNA-Thr-ED domain became fused to SepRS may be because bacteria, unlike archaea, possess tRNA^Gly^ with U73 (Hohn et al., 2006). Thus, the addition of tRNA-Thr-ED to the bacterial SepRS-SepCysS system appears to impose a restriction on tRNA-Thr-ED hydrolytic activity, as achieved by fusion with SepRS.

### 3.3 Non-Canonical and Degenerated Homologs of SepRS

#### 3.3.1 SepRS-like Proteins From Thermoplasmata Archaea May Not Aminoacylate Sep

**FIGURE 6:**
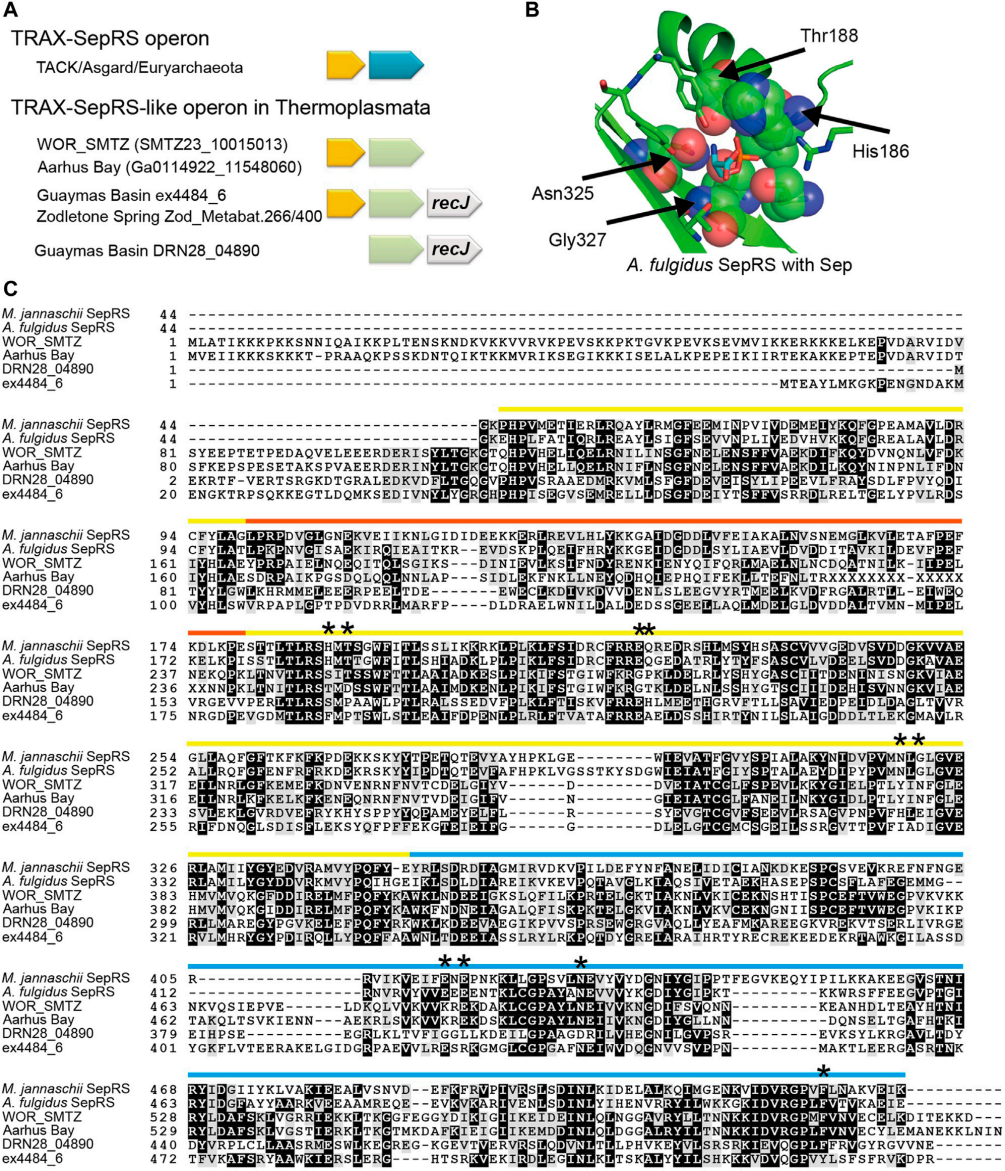
Characterization of SepRS-like proteins from two lineages of Thermoplasmata archaea **(A)** The operon structures of the four SepRS-like genes identified. Like many SepRS genes, three SepRS-like genes are headed by a TRAX homolog gene. In the Guaymas Basin hydrothermal vent lineage and the Zodletone Spring lineage, the SepRS-like genes are followed by a predicted *recJ* gene **(B)** The four residues important for Sep recognition by SepRS are altered in SepRS-like proteins **(C)** Multiple sequence alignment of SepRS-like proteins with two reference SepRS proteins. Interesting mutations are denoted by stars. The color bars represent the domain structure and follow the same scheme as in Figure 3, with the yellow bar denoting the catalytic domain, the orange bar denoting the inserted domain, and the blue bar denoting the anticodon binding domain. A part of the Aarhus Bay SepRS-like sequence remains elusive (indicated with Xs).

Two lineages of Thermoplasmata archaea living in deep subsurface microbial communities, marine hydrothermal vents, and hot springs (Dombrowski et al., 2018; Hahn et al., 2021) encode SepRS-like proteins (Figure 4A and Figure 6). According to our analyses, both the SepRS and SepCysS encoding sequences are absent in all other Thermoplasmata members. Interestingly, similar to many SepRS genes, the SepRS-like genes form an operon together with a TRAX gene (Gupta et al., 2012) (Figure 6A). Most likely, the common ancestor of Thermoplasmata has repurposed its TRAX-SepRS operon. Further analysis of the SepRS-like sequence revealed nonsynonymous exchanges in the Sep-accommodating pocket, indicating that these proteins may not recognize Sep (Figure 6B). Indeed, the perfectly conserved Asn-X-Gly motif of SepRS in the *ß*-sheet is replaced by Tyr-Ile-Asn, His-Leu-Glu or Ile/Leu-X-Asp (Figure 6C). These same residues were critical in altering the substrate specificity of SepRS for synthetic biology purposes, allowing the engineered variant to incorporate non-canonical amino acids other than Sep (Zhang et al., 2017). Furthermore, the annotation of SepRS-like species belonging to the marine subsurface clade suggests the presence of an additional N-terminal domain, which appears to be a DNA/RNA-binding domain, although we cannot exclude the possibility of misannotation of the start codon. Interestingly, the catalytic *α*-subunit of archaeal/eukaryotic PheRS (PheRSα) has a similar configuration with an N-terminal DNA-binding module appended to the aminoacylation domain (Finarov et al., 2010). Since SepRS is thought to have evolved from PheRSα (Kamtekar et al., 2007), this similarity may support the reading frame annotation.

**FIGURE 7:**
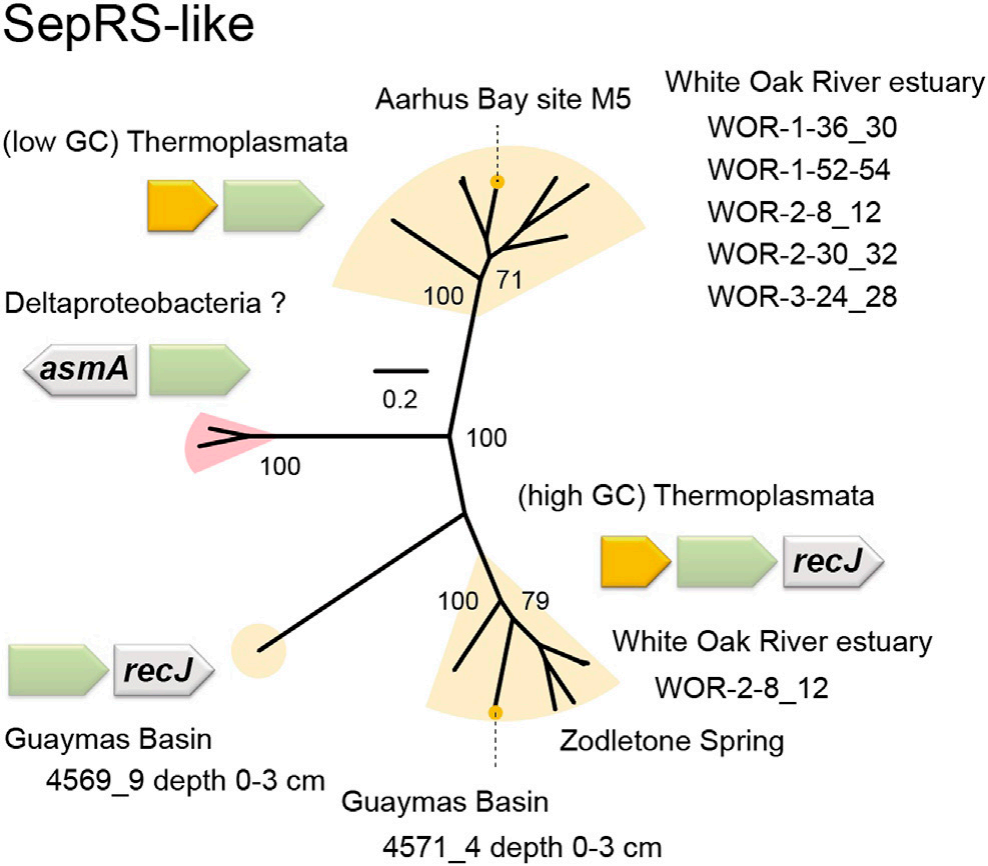
Phylogenetic tree and operon structures of SepRS-like homologs. This unrooted tree was made by maximum likelihood estimation with 100 replicates. The bootstrap values (percentages) are given. The metagenomic origins are shown for each clade. The (low GC) Thermoplasmata SepRS-like genes are headed by a TRAX homolog gene (yellow), while the (high GC) Thermoplasmata SepRS-like genes are followed by a *recJ* gene. The novel SepRS-like group (pink) may belong to Deltaproteobacteria, because their neighboring genes show high similarities to Deltaproteobacteria genes. The *asmA* gene may encode the bacterial outer membrane protein assembly protein AsmA.

Manual analysis of Sequence Read Archive (SRA) data of microbial communities in marine sediments from the metagenomes of the White Oak River estuary (WOR) revealed further examples of both types of Thermoplasmata SepRS-like genes (Figure 7, Supplementary Table S1). One of them belongs to Thermoplasmata with low GC content (<40%), while the other belongs to Thermoplasmata with high GC content (>40%) (Figure 7). Surprisingly, a new group of SepRS-like genes was found in one of the WOR metagenome datasets (WOR-2–8_12), which probably belong to Deltaproteobacteria (Figure 7, Supplementary Table S1). The Asn-X-Gly motif of SepRS is altered in these putative bacterial SepRS-like genes to Tyr-Leu-Ser, but overall, they did not show significant similarity to those of Thermoplasmata. It can be speculated that the SepRS-like proteins attach an amino acid other than Sep to a tRNA. On the other hand, these enzymes may mediate a tRNA-utilizing reaction other than aminoacylation, which would suggest an alternative physiological role in some marine lineages of Thermoplasmata and Deltaproteobacteria.

#### 3.3.2 Occurrence of SepRS Domains and SepRS-like Proteins in Micrarchaeota Groups

**FIGURE 8:**
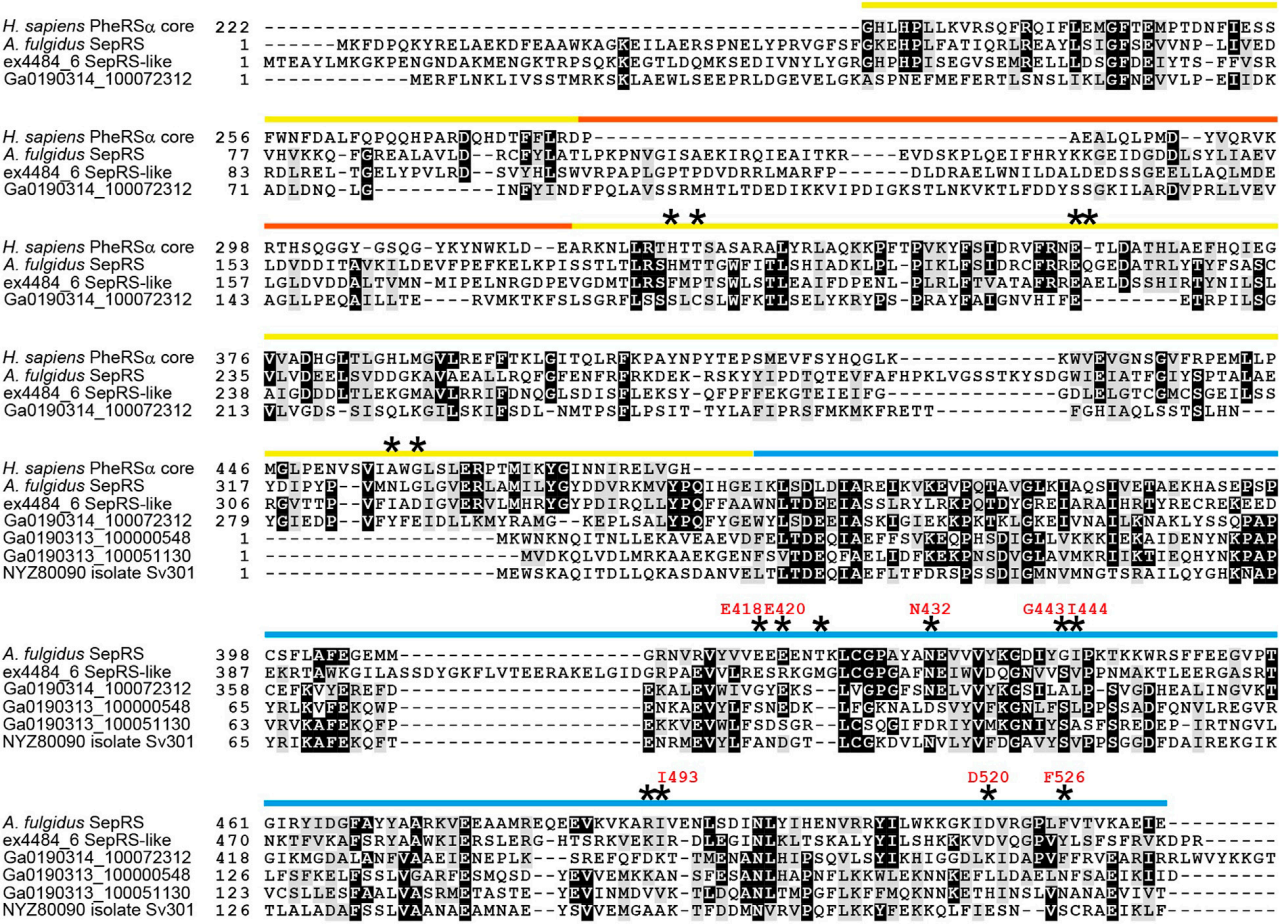
Characterization of degenerated SepRS homologs from Alv-FOS5/pISA35 groups of Micrarchaeota archaea. Multiple sequence alignment of SepRS homologs with the catalytic core of *Homo sapiens* PheRS (residues 222-480). Interesting mutations are indicated with stars. The amino acid residues of *A. fulgidus* SepRS which are important for anticodon loop recognition are indicated with red characters. The color bars represent the domain structure and follow the same scheme as in Figure 3, with the yellow bar denoting the catalytic domain, the orange bar denoting the inserted domain, and the blue bar denoting the anticodon binding domain. The full-length SepRS homolog belongs to a putative Alv-FOS5-group Micrarchaeota archaeon (Ga0190314_1000723, Ga0190313_1000196, Ga0190313_1000256, Ga0190313_1002238), while the small homologs belong to pISA35-group Micrarchaeota archaea (Ga0190313_1000005, Ga0190313_1000009, Ga0190313_1000511, Ga0190313_1005305) in Guaymas Basin hydrothermal vent metagenomes (4870-07-11-12_MG and 4870-07-10-11_MG) and in a freshwater sediment metagenome from Lake Svetloe, Arkhangelsk region (PRJNA644262).

Another SepRS-like homolog was found in a metagenomic bin of a Micrarchaeota Alv-FOS5-group (Moussard et al., 2006) archaeon whose sequence was assembled from Guaymas Basin hydrothermal vent metagenomes (Figure 4A and Figure 8). No other example was found in the public genome/metagenome sequence repositories we examined, probably due to the limited distribution of this archaeal group within populations of particular hydrothermal vents (Moussard et al., 2006; Dombrowski et al., 2018; Vázquez-Campos et al., 2021). The sequence of the Alv-FOS5-group homolog is highly degenerated with multiple mutations and small insertions and deletions (Figure 8). Curiously, we found homologs of the C-terminal anticodon-binding domain of SepRS in the metagenomic bins of two putative archaea belonging to the Micrarchaeota pISA35-group (Figures 4A and Figure 8). The two small SepRS homologs are highly diverged from each other but remain associated with an asparaginyl-tRNA synthetase gene (*asnS*) in the opposite direction (Figure 4A). It is plausible to assume that these degenerate SepRS homologs perform functions that support other aminoacylation systems, as all twenty aaRSs appear to be present in these organisms.

### 3.4 Diversity of the Archaeal Selenocysteine-Encoding System

The crystal structure of the transsulfursome complex shows that the tetrameric SepRS is positioned in the center, with two SepCysE adapters mediating contact with two dimeric SepCysS molecules (Figure 3). A subcomplex consisting of SepCysS, SepCysE, and tRNA^Cys^ shows that SepCysS utilizes the SepRS determinant U73, whereas the C-terminal domain of SepCysE binds tRNA^Cys^ nonspecifically (Chen M. et al., 2017). Our earlier study indicated that indirect systems for encoding Cys and Sec may have co-evolved (Mukai et al., 2017a). As mentioned previously, Sec-utilizing archaeal organisms possess SepCysE, the adapter essential for effective shuttling of Sep-tRNA^Cys^ between SepRS and SepCysS. In the absence of SepCysE, and thus the transsulfursome, there is a possibility that SepCysS accepts an intermediate of the Sec pathway: although *in vitro* data show that the tRNA^Cys^ determinant U73 is stringently recognized, archaeal SepCysS *in vivo* is known to accept the G73-containing Sep-tRNA^Sec^ instead (Yuan et al., 2010). This crosstalk may have deleterious consequences, as substitution of Cys for Sec can result in proteins with altered activity (Hondal et al., 2013).

#### 3.4.1 Absence of the Adapter Protein SepCysE in Bathyarchaeota and Archaeoglobi Is Associated With Idiosyncratic Changes in the Sec-Encoding Machinery

**FIGURE 9:**
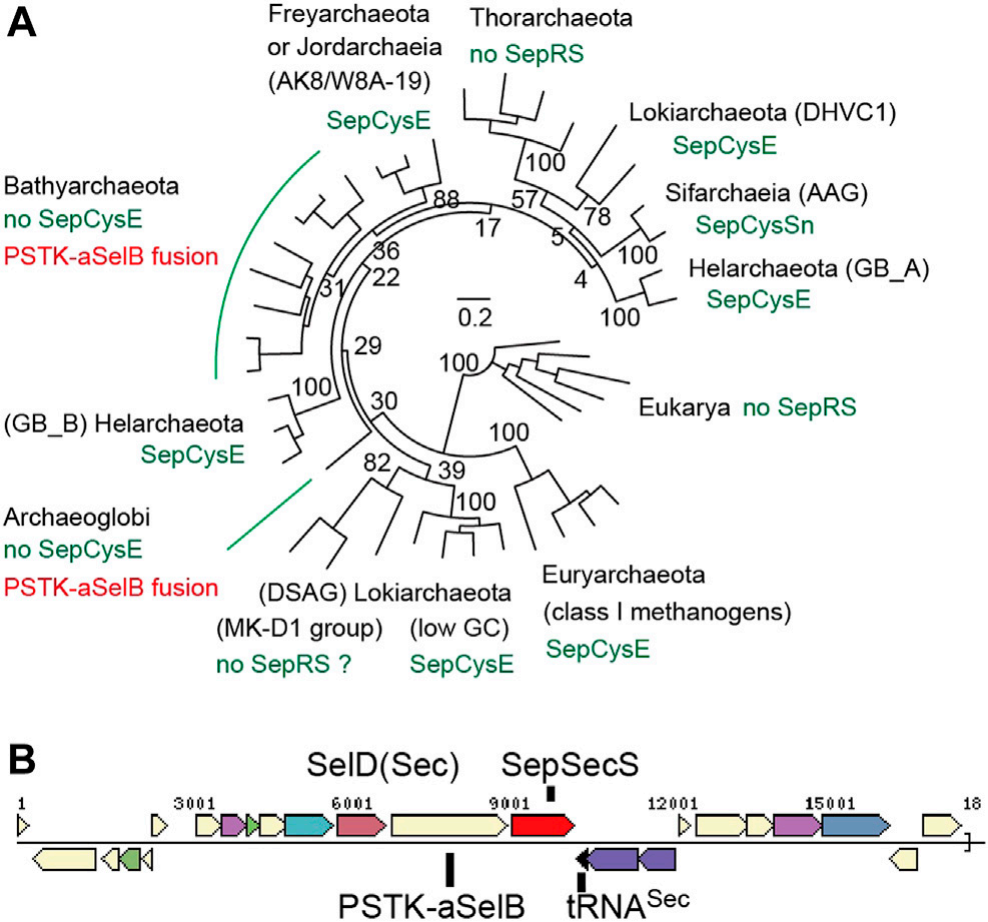
Distribution and classification of Sec-encoding systems in archaea **(A)** A SepSecS phylogenetic tree with bootstrap values (percentages) made by maximum likelihood estimation with 100 replicates is shown **(B)** A typical gene cluster for Sec encoding and utilization in Bathyarchaeota (3300008019.a:Ga0105158_100044113).

Figure 9A, Supplementary Figure S2 show the updated phylogenetic tree of Sep-tRNA:Sec-tRNA synthase, SepSecS (Palioura et al., 2009). Consistent with our earlier findings, Sec-utilizing archaea with indirect Cys encoding pathways possess the SepCysE adapter protein or its N-terminal domain (the ‘SepCysSn’ peptide). The only exceptions are some lineages of Bathyarchaeota and Archaeoglobi, which also harbor some highly divergent Sec-encoding systems. Diverse Bathyarchaeota and one Archaeoglobus species contain an atypical Sec-encoding system, in which PSTK and the Sec-specific elongation factor SelB (aSelB) are fused within a single open reading frame (ORF) (Figures 9A,B). Our annotation indicates that this Archaeoglobus species has both a Sec-encoding and a SepRS-SepCysS system, the latter lacking the SepCysE adapter. This suggests that the unusual PSTK-aSelB fusion protein may serve to protect Sep-tRNA^Sec^ from being erroneously recognized by SepCysS (Yuan et al., 2010).

#### 3.4.2 Idiosyncrasies of Other Sec- and Two-step Cys-Encoding Systems

Interestingly, in a small lineage of Sifarchaeia archaea (Sun et al., 2021) possessing SepRS-SepCysS-SepCysSn, their PSTK and aSelB ORFs are split by a stop codon (Figure 9A). Thus, erroneous conversion of Sep-tRNA^Sec^ to Cys-tRNA^Sec^ might be acceptable to these archaea to some extent. Bacterial and eukaryotic systems are known to misincorporate Cys at Sec positions and *vice versa* (Turanov et al., 2009; Xu et al., 2010; Mukai et al., 2016; Vargas-Rodriguez et al., 2018). Another similarity between this and eukaryotic Sec-encoding systems is that wetland sediment lineage of Bathyarchaeota has tRNA^Sec^ with a U6-U67 mismatch (Supplementary Figure S3). This feature, which is conserved in eukaryotic tRNAs^Sec^ (Sherrer et al., 2008), together with the possible Sec/Cys cross-talk in Sifarchaeia, indicates that the eukaryotic Sec system may have evolved from an ancient Bathyarchaeota/Asgard group (Figure 9A).

## 4 Discussion

All indirect acylation systems are ancient and are considered to have been present at the time of the last universal common ancestor (LUCA) (O'Donoghue et al., 2005; Yuan et al., 2006; Sheppard and Söll, 2008). These include Cys- and both bacterial and archaeal/eukaryotic Sec-tRNA biosynthetic pathways (Yuan et al., 2006). It is likely that in LUCA only indirect pathways served to form Gln-tRNA^Gln^ and Asn-tRNA^Asn^, whereas GlnRS and AsnRS evolved later. Although a substantial number of prokaryotes have direct Gln and Asn acylation systems, the vast majority rely on indirect acylation systems for the biosynthesis of Asn-tRNA (most bacteria and archaea) and Gln-tRNA (most bacteria and almost all archaea). The absence of GlnRS in archaea may be due to idiosyncratic features of archaeal tRNA^Gln^, which is considered orthogonal to bacterial and eukaryotic GlnRS (Tumbula et al., 2000). Thus, while bacteria can encode any combination of the indirect and direct Asn-tRNA and Gln-tRNA pathways, Gln-tRNA formation in archaea always occurs via the indirect pathway, whereas Asn-tRNA biosynthesis can proceed in a direct or indirect manner. It is important to note that in certain bacteria, tRNA-dependent biosynthesis of Asn is also the sole route to Asn (Min et al., 2002); such organisms do not possess asparagine synthetase (encoded by *asnA* or *asnB*), which normally generates free Asn without tRNA involvement. In such cases, both the direct and indirect Asn-tRNA acylation systems may coexist in the organism, with the main role of the indirect acylation system being the biosynthesis of Asn (Becker and Kern, 1998; Min et al., 2002).

Similarly, organisms possessing both SepRS and CysRS may rely predominantly on the former for Cys biosynthesis, as deletion of a transsulfursome component leads to Cys auxotrophy (Sauerwald et al., 2005; Liu et al., 2014). The redundancy of direct and indirect pathways allowed the evolution of organisms that support their demand for Asn-, Cys-, or Gln-tRNA by encoding the direct or indirect pathway, or a sometimes a combination of both. The reasons for an organism’s selection of the route to aa-tRNA is unknown; the organism’s metabolism, metabolome, and habitat may be important elements. For example, the presence of a tRNA-dependent Cys biosynthetic pathway may be related to the high demands of the organism, as methanogens have almost twice as much Cys in their proteins compared to other archaea (Klipcan et al., 2008); similarly, the only methanogens that do not use the indirect Cys encoding systems are those that live in sulfide-deficient environments, as in the case of the methanogens of the human gut microbiome (Dridi et al., 2011).

Both the SepRS- and CysRS-dependent Cys-tRNA pathways are thought to be ancient and present at the time of LUCA (O'Donoghue et al., 2005). CysRS was then only retained in species that later differentiated into bacterial lineages. Present-day methanogens possessing CysRS are thought to have acquired it from bacteria through horizontal transfer at later stages (O'Donoghue et al., 2005). Importantly, the two aaRSs share the same identity determinants (the anticodon, U73 discriminator, and the methylated base G37). In methanogens where both direct and indirect route enzymes are present, SepRS and CysRS can acylate a single tRNA (e.g. in *Methanosarcina acetivorans*, *Methanococcus maripaludis*) or preferentially accept one or two tRNA isoacceptors (e.g. *Methanosarcina mazei*) (Hauenstein and Perona, 2008). On the other hand, CysRS may have co-evolved with the SepRS acylation system in diverse SepRS-utilizing methanogens (Perona et al., 2018). Some *Methanospirillum* and *Methanoregula* species possess a G73-containing tRNA^Cys^, whereas some *Methanobacterium* species possess C73. An A73-containing tRNA^Cys^ is found in *Methanolobus* and *Methanocella* species (Perona et al., 2018). Interestingly, these changes in the discriminator base are accompanied by mutations in the acceptor stem-binding region of CysRS, suggesting that the SepRS and CysRS systems may be orthogonal in these organisms or that some tRNAs are preferentially recognized by either SepRS or CysRS. The noncanonical features of these tRNAs could also allow these molecules to escape tRNA-Thr-ED editing.

The results of this study provide new information on the evolution and distribution of the two minor genetic code systems. Obscure lineages of thermophilic archaea appear to have inherited ancient forms of these systems, possibly over several billion years. We have previously found that Sec-utilizing organisms that rely on indirect Cys-tRNA biosynthesis contain the adapter protein SepCysE; SepCysE likely serves to prevent cross-talk between these two systems. Here, we identified lineages of Bathyarchaeota and Archaeoglobi in which SepCysE is absent, but contain a PSTK-aSelB fusion that may serve the same function. Conversely, a SepRS-dependent Cys-encoding system is present in Sifarchaeia archaea that have neither SepCysE nor a PSTK-aSelB fusion, indicating that some crosstalk between Sec and Cys systems may occur in these organisms, similar to some bacterial and eukaryotic systems. However, many questions remain to be investigated. 1) Are these systems a late invention of the genetic code? Or were they renovated and modernized from their primitive forms in some archaeal lineages? 2) Did the two Sep-tRNA-mediated systems (Cys- and Sec-coding) co-evolve in archaea to prevent their crosstalk? 3) What functions might the non-canonical SepRS homologs have? To answer these questions, we need more genomic and metagenomic data from archaea living in the deep marine subsurface and/or in hydrothermal vents to fill in the gaps. This would allow us to find ancient SepRS genes that have been inherited in obscure archaeal lineages. To build more reliable phylogenetic trees of genes, a more reliable evolutionary tree of archaea is also a prerequisite.

Deciphering the distribution and diversity of Cys and Sec encoding systems in obscure, uncultured microbes is important in several respects: 1) the SepRS-SepCysS system is important for sulfur assimilation and implicated in biological activities such as methanogenesis, methylamine metabolism, and organohalide respiration that may have global impact on the Earth, 2) uncultured bacteria and archaea provide an expanded record of the evolution of the genetic code, 3) as SepRS is invaluable for genetic code expansion (Park et al., 2011; Zhang et al., 2017; Beranek et al., 2018), naturally diverse SepRS species could inspire further applications in the field of synthetic biology (Mukai et al., 2017b). The knowledge may help us speculate about the early evolution of the standard genetic code and give us a hint for developing a new orthogonal aminoacylation system (Cervettini et al., 2020) by rational design.

## Data Availability

The datasets presented in this study can be found in online repositories. The names of the repository/repositories and accession number(s) can be found in the article/Supplementary Material.
