# Supplementary material for: Indirect Routes to Aminoacyl-tRNA: The Diversity of Prokaryotic Cysteine Encoding Systems
Source: Front Genet. 2022 Jan 3;12:794509. doi: 10.3389/fgene.2021.794509 (PMC8762117; doi:10.3389/fgene.2021.794509)
Supplement: Supplementary file 1 [file DataSheet1.docx]

Supplementary Material

# Supplementary Information

## Materials and Methods

### Bioinformatics

*Differences from our previous analysis* [1]**.** Some of the SepRS and SepCysS sequences derived from metagenomic contigs were replaced with sequences which were recently deposited by others to the NCBI database as composite genome or single cell genome. Phylogenetic inference of archaeal and bacterial species was performed by using the SILVA ribosomal RNA gene databases [2]. Most of unknown Miscellaneous Crenarchaeota group (MCG) archaea were thus annotated to belong to Bathyarchaeota. Although the IAN1-71 archaea were annotated to use SepRS and SepCysS in our previous analysis, these SepRS-SepCysS genes were revealed to belong to a group of Bathyarchaeota. The unknown “BOG (Asgard) archaeon” and “WOR (Asgard-like) archaeon” are now annotated to belong to Lokiarchaeota and Sifarchaeia, respectively. AK8/W8A-19 Bathyarchaeota are now annotated as Asgard Freyarchaeota or Jordarchaeia. We also corrected a few mis-annotations in our previous paper [1]. The SepRS-SepCysS metagenomic contig of “Crystal Geyser bacterium No. 3” is now annotated to belong to a Woesearchaeon by considering the genomic GC content, whereas the contig has a part of a deltaproteobacterial transposon. The SepRS sequence of “GB archaeon No. 2” provided in our previous supplementary file had a one-residue deletion. New SepRS, SepCysS SepCysE, SepCysSn, and SepSecS sequences were obtained and analyzed by the same method [1].

- The SepRS-like protein sequence of the Aarhus_00045 Thermoplasmata species was speculated by manually assembling a metagenomic bin using the following sequence data (3300011118.a:Ga0114922_10404840, _11548060, _10325614, gnl|SRA|SRR6056591.104879282.2, .22125619.2, .23746657.2, .37729466.1, 110458889.1, .97071966.2, .23746657.1, .110458889.1).
- Two high GC Lokiarchaeota species were found in the Bog ECP12_OM1 metagenome datasets. Their putative 16S rRNA genes are 3300027825.a:Ga0209039_100037679 and 3300004152.a:Ga0062386_1003294631.
- A small group of Sifarchaeia was found in the White Oak River estuary, Sumatra_00607, and Benguela_00093 metagenome datasets.

# Supplementary Tables and Figures

## Supplementary Table

**Table S1** List of metagenomic datasets mainly used for this study.

- Bog forest soil microbial communities from Calvert Island, British Columbia, Canada - ECP12_OM1 (SPAdes)
- Groundwater microbial communities from aquifer - Crystal Geyser CG07_land_8/20/14_0.80
- Marine sediment microbial communities from White Oak River estuary, North Carolina - WOR_SMTZ
- Groundwater microbial communities from the Aspo Hard Rock Laboratory (HRL) deep subsurface site, Sweden
- Sorted cell/s from the Aspo Hard Rock Laboratory (HRL) deep subsurface site groundwater, Oskarshamn, Sweden
- Sorted cell/s from groundwater in Star Diamonds mine, Free Sate, South Africa
- Sediment microbial communities from Lake Kivu, Rwanda
- Anoxic lake water microbial communities from Lake Kivu, Rwanda to study Microbial Dark Matter (Phase II)
- Hot spring microbial mat communities from California, USA to study Microbial Dark Matter (Phase II) - Cone Pool mat layer H metaG (SPAdes)
- Deep subsurface microbial communities from Aarhus Bay to uncover new lineages of life (NeLLi) - Aarhus_00045
- Deep subsurface microbial communities from Anholt, Denmark to uncover new lineages of life (NeLLi) - Anholt_01485
- Deep subsurface microbial communities from Black Sea to uncover new lineages of life (NeLLi) - Black_00105
- Deep subsurface microbial communities from Indian Ocean to uncover new lineages of life (NeLLi) - Sumatra_00157
- Deep subsurface microbial communities from Indian Ocean to uncover new lineages of life (NeLLi) - Sumatra_00607
- Deep subsurface microbial communities from South Pacific Ocean to uncover new lineages of life (NeLLi) - Chile_00310
- Deep subsurface microbial communities from South Atlantic Ocean to uncover new lineages of life (NeLLi) - Benguela_00093
- Hydrothermal vent sediment bacterial communities from Southern Trench, Guaymas Basin, Mexico
- Subseafloor sediment microbial communities from Guaymas Basin, Gulf of California, Mexico
- Hydrothermal vent microbial communities from East Pacific Rise, Pacific Ocean
- Hydrothermal vent microbial communities from Mid Atlantic Ridge, Atlantic Ocean
- Kivu_combined assembly
- Boni_combined assembly
- Guaymas_combined assembly
- anaerobic digester metagenome (Accession: PRJNA321808)
- Active sludge microbial communities of municipal wastewater-treating anaerobic digesters from Japan - AD_JPNHW2_MetaG and USA - AD_UKC030_MetaG
- Archaeal genomes from LFLS groundwater (PRJEB14718)
- Metagenome-assembled genomes from hydrothermal vent and freshwater spring sediments (PRJNA480137)
- Collection of 3,087 bacterial metagenome-assembled genomes recovered from metagenomes available from the Sequence Read Archive (PRJNA417962)
- hot springs metagenome (PRJNA392119)
- anoxic spring sediments of Zodletone spring in southwestern Oklahoma
- metagenome-assembled genomes of Asgardarchaeota obtained from habitats around the globe - BioProjects: PRJNA678545 (Sunshine Coast lakes), PRJNA678552 (Hikurangi Subduction Margin) and PRJNA678817 (newly assembled and binned genome sequences from public metagenomes)

## Supplementary Figures


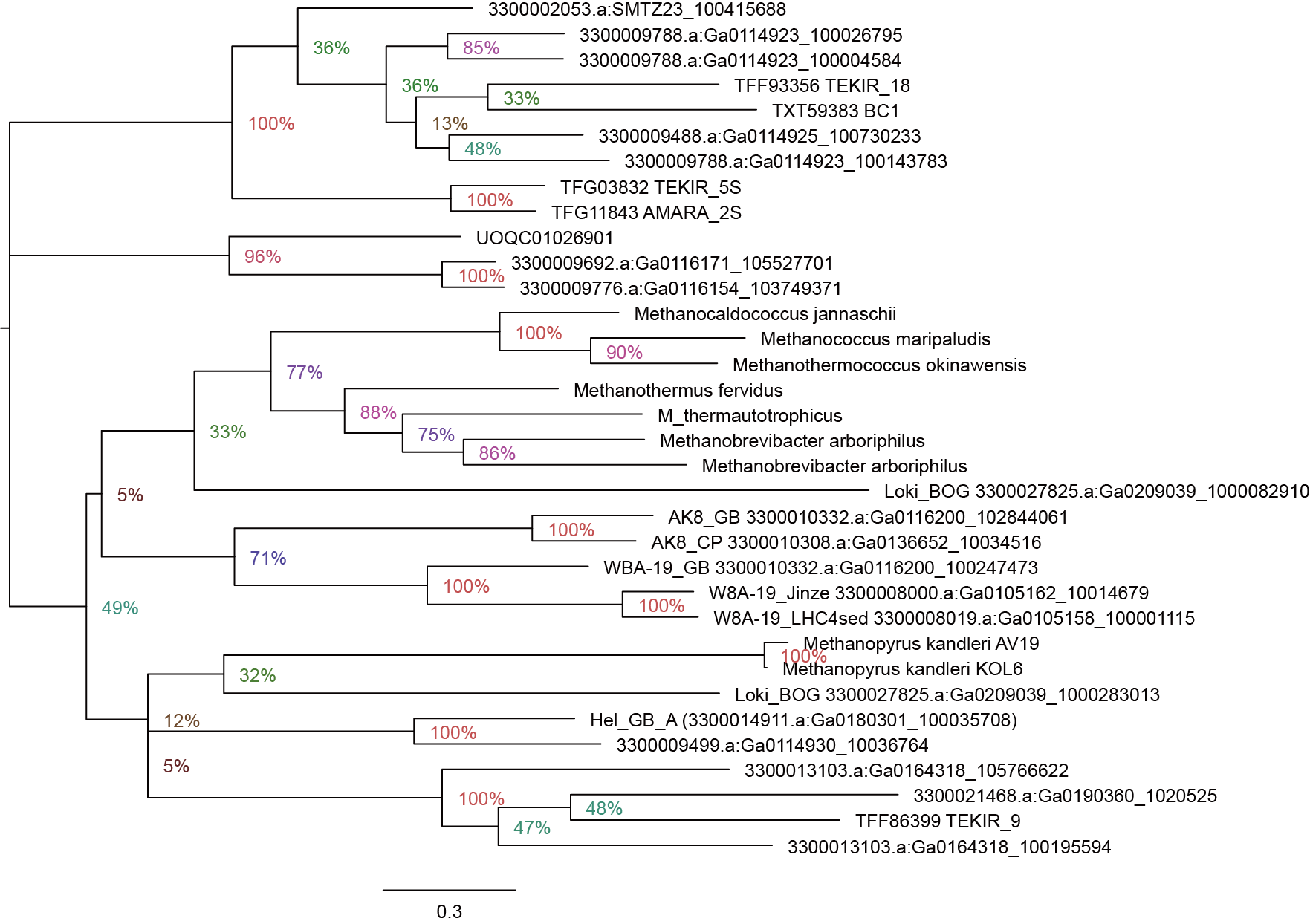


**Supplementary Figure 1.** The uncompressed SepCysE phylogenetic tree of Fig. 4C.


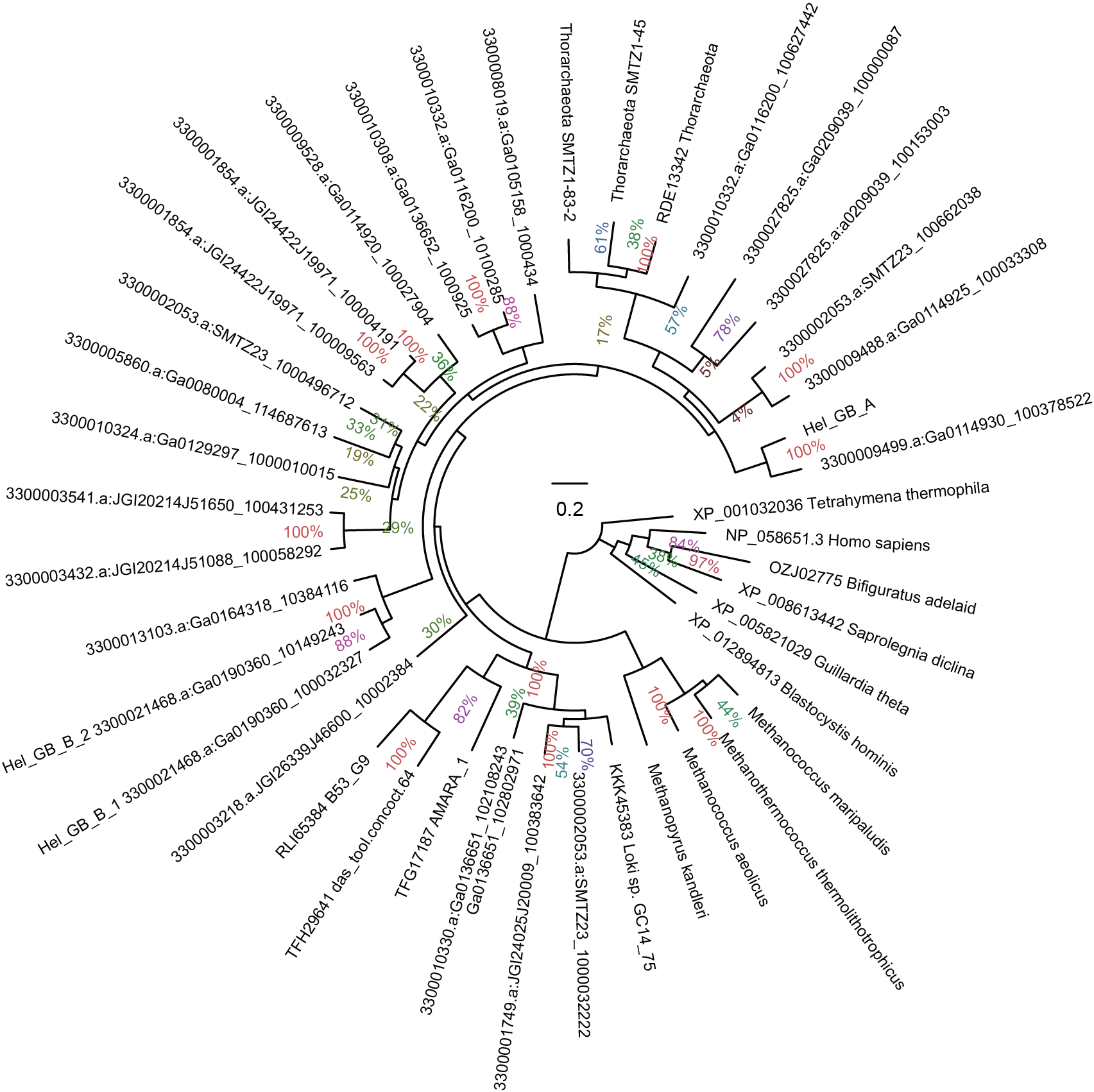


**Supplementary Figure 2.** The uncompressed SepSecS phylogenetic tree of Fig. 9.

**Supplementary Figure 3.** Archaeal tRNA^Sec^ sequences having a U6-U67 mismatch in the acceptor stem.
